# Repeat dose exposure of PM_2.5_ triggers the disseminated intravascular coagulation (DIC) in SD rats

**DOI:** 10.1016/j.scitotenv.2019.01.346

**Published:** 2019-05-01

**Authors:** Shuang Liang, Tong Zhao, Hejing Hu, Yanfeng Shi, Qing Xu, Mark R. Miller, Junchao Duan, Zhiwei Sun

**Affiliations:** a Department of Toxicology and Sanitary Chemistry, School of Public Health, Capital Medical University, Beijing 100069, PR China; b Beijing Key Laboratory of Environmental Toxicology, Capital Medical University, Beijing 100069, PR China; c Core Facility Centre, Capital Medical University, Beijing 100069, PR China; d University/BHF Centre for Cardiovascular Science, Queens Medical Research Institute, The University of Edinburgh, Edinburgh, UK

**Keywords:** PM_2.5_, Inflammation, Endothelial injury, Coagulation disorder, Disseminated intravascular coagulation (DIC)

## Abstract

Epidemiological evidence suggests that fine particulate matter (PM_2.5_) in air pollution promotes the formation of deep venous thrombosis. However, no evidence is available on the effects of PM_2.5_ lead to disseminated intravascular coagulation (DIC). For the first time, this study explored the effects of PM_2.5_ on DIC *via* coagulation disorders *in vivo*. SD rats received intratracheal instillation of PM_2.5_ once every three days for one month. Doppler ultrasound showed that the pulmonary valve (PV) and aortic valve (AV) peak flow were decreased after exposure to PM_2.5_. Fibrin deposition and bleeding were observed in lung tissue and vascular endothelial injury was found after exposure to PM_2.5_. Expression of thrombomodulin (TM) in vessel was downregulated after PM_2.5_-treated, whereas the levels of proinflammatory factors and adhesion molecules (IL-6, IL-1β, CRP, ICAM-1 and VCAM-1) were markedly elevated after exposure to PM_2.5_. Tissue factor (TF) and the coagulation factor of FXa were increased, while vWF was significantly lowered induced by PM_2.5_. Thrombin-antithrombin complex (TAT) and fibrinolytic factor (t-PA) were elevated, while there was no significantly change in the expression of anticoagulant factors (TFPI and AT-III). To clarify the relationship between PM_2.5_ and DIC, we examined the general diagnostic indices of DIC: PM_2.5_ prolonged PT and increased the expression of D-dimer but decreased platelet count and fibrinogen. In addition, the gene levels of JAK1 and STAT3 showed an upward trend, whereas there was little effect on JAK2 expression. And inflammatory factors (IL-6, IL-1β and TNF) in blood vessels of were up-reglated in PM_2.5_-treated rats. In summary, our results found that PM_2.5_ could induce inflammatory response, vascular endothelial injury and prothrombotic state, eventually resulted in DIC. It will provide new evidence for a link between PM_2.5_ and cardiovascular disease.

## Highlights

• PM_2.5_ triggered vascular endothelial injury *via* inflammatory response.
• PM_2.5_ activated TF-dependent coagulation and caused coagulation dysfunction.
• PM_2.5_ induced fibrin deposition and bleeding in pulmonary and resulted in DIC.

## Graphical abstract

**Figure:**
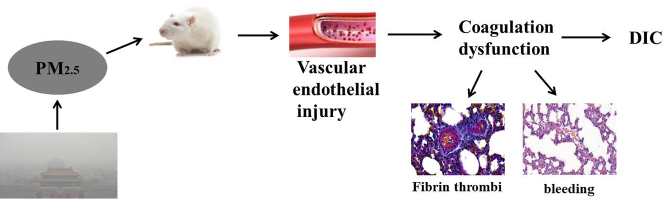

## 1 Introduction

The Global Burden of Disease (GBD) consortium reported that the mortality association with air pollution is a primarily attributed to cardiovascular disease (Landrigan et al., 2018). The WHO estimated that outdoor air pollution causes 4.2 million premature deaths worldwide in 2016. The American Heart Association (AHA) estimates that cardiovascular diseases will remain the major cause for premature deaths from air pollution for the next twenty years (Brauer et al., 2016).

Thrombosis plays a significant role in cardiovascular mortality, and there is a growing body of evidence that air pollution promotes an imbalance in thrombotic, coagulation and fibrinolytic pathways (Robertson and Miller, 2018). Epidemiological evidence showed that particulate air pollution has an effect on the coagulability of blood, increasing the susceptibility of individuals to acute episodes of cardiovascular disease (Seaton et al., 1995; Seaton et al., 1999). Baccarelli et al. also reported that every 10 μg/m^3^ increase in PM was associated with a 70% increase in deep venous thrombosis (DVT) (Baccarelli et al., 2008). Thrombotic pathways are also important pathological mechanisms for the occurrence of such severe coagulation disorders, disseminated intravascular coagulation (DIC) (Okamoto et al., 2016).

Several clinical conditions, in particular those associated with a systemic inflammatory response, can lead to activation of coagulation but when the procoagulant is uncontrolled and overcomes the natural anticoagulant mechanisms of coagulation, DIC may occur (Levi, 2018). DIC is characterized by a widespread activation of coagulation, which leads to microvascular fibrin deposition of the liver, kidney, brain, especially in lung and then development of multi-organ failure (Gando et al., 2016; Kaufman et al., 1984). Bleeding then follows contributes to platelet and coagulation factors consumption as well as platelet dysfunction during the cause of DIC (Laursen et al., 2018). The general diagnostic indices of DIC: platelet count, prothrombin time, fibrinogen levels, and fibrin-related markers, fibrin deposition is very important lab pathological evidence of DIC (Berthelsen et al., 2011; Tsantarliotou et al., 2013). Epidemiologic studies indicated that exposure to PM_2.5_ induced endothelial activation by enhancing the expression of pro-inflammatory factors and TF, leading to coagulation and fibrinolysis (Hajat et al., 2015; Viehmann et al., 2015). There are relatively few studies using *in vivo* models of thrombosis to address the mechanistic pathways induced by air pollution, with no study looking at potential relationship between PM_2.5_ and DIC.

Therefore, the following scientific hypothesis in this study was proposed: exposure of rats to PM_2.5_ induces dysfunction of coagulation by activating the TF-dependent pathways, simultaneously with t-PA-mediated secondary hyperfibrinolysis, leading to DIC. In this study, we investigated the levels of inflammation and coagulation factors after exposure to PM_2.5_. Vascular endothelial injury was demonstrated by immunohistochemical and HE stain. Fibrin deposition and bleeding were observed by MSB or HE stains in lungs of rats. The findings supported this hypothesis providing compelling new mechanistic evidence for a link between PM_2.5_ and cardiovascular morbidity and mortality.

## 2 Materials and methods

### 2.1 PM_2.5_ preparation and characterization

PM_2.5_ was sampled from the roof of Capital Medical University building in central Beijing, China in the winter of 2016. Samples were collected onto the new quartz fiber filters (8 × 10 in. Pall, USA) with a large-volume air particle sampler at a constant flow rate of 1.05m^3^/min (TH-1000CII, Wuhan Tianhong, China). Filters with PM_2.5_ were cut into small pieces and transferred them into ultrapure water, and followed by sonication for three hours in 4 °C. The collected suspension was freeze-dried using a vacuum freeze dryer (ALPHA 1-2LD PLUS, Marin Christ, Germany). After two hours' ultraviolet radiation, the dried samples were diluted and mixed with sterilized saline, and then sonicated for 30 min to resuspend the PM_2.5_.

The major chemical components of PM_2.5_ have been detail described in our previous study, and they come from the same batch at a same time point. The average concentrations of OC and EC in PM_2.5_ were 222.27 ± 35.55 mg/g and 47.97 ± 42.46 mg/g, respectively. Among the total of 28 inorganic elements measured in the PM_2.5_, especially S, Si, K and Fe were the most abundant elements. Toxic heavy metals (including Pb, Mn, Cd, Cr and Ni) and toxic nonmetallic elements (As) were both detected in PM_2.5_. The total PAHs concentration was 1042.80 ± 474.76 μg/g (Y. Zhang et al. 2017).

### 2.2 Animals and exposure to PM_2.5_

Specific pathogen free (SPF) male Sprague-Dawley (SD) rats (8–12 weeks old) were obtained from the Beijing Vital River Laboratory Animal Technology Co., Ltd. (Beijing, China). The Animal Experiments and Experimental Animal Welfare Committee of Capital Medical University approved ethical requirements (permit number: AEEI-2016-076). Rats were randomly divided into four groups (*n* = 6 rats/group) control, low, middle and high doses of PM_2.5_ (0, 1.8, 5.4 and 16.2 mg/kg bw, respectively). Rats received intratracheal instillation of PM_2.5_ under anesthesia (5% chloralhydrate, 7 ml/kg bw) every 3 days for 30 days. Animals were euthanized immediately after exposure to PM_2.5_.

The installation doses of PM_2.5_ were based on physiological parameters of rats and the World Health Organization air quality guidelines (WHO, 2006): The respiratory volume of an adult rat (200 g) is 0.86 ml/each breath, the breath rate is 85 times/min. According to the annual mean concentrations of interim target-1 of PM_2.5_ (35 μg/m^3^) recommended by the WHO, the amount of PM_2.5_ exposure of one day is 3.684 μg. After a 100-fold uncertainty factor was applied, the concentration of PM_2.5_ every day to be exposed was determined to be 1.8 mg/kg bw. Based on the concentration of 1.8 mg/kg bw used as a low-dose, a 3-fold (5.4 mg/kg bw) and a 9-fold (16.2 mg/kg bw) concentration were used as moderate- and high-dose, respectively (Y. Zhang et al. 2017; Duan et al., 2017).

### 2.3 PM_2.5_ endotoxins analysis

The level of PM_2.5_ endotoxins was detected using Rapid Gel Clot TAL Endotoxin Test Kit (Xiamen Bioendo Technology, China). The limit of detection is 0.25EU/ml. The resuspended PM_2.5_ was sonicated for 30 min, and centrifuged at 3000r for 15 min and the supernatant was transferred to a pyrogen-free test tube. The samples were diluted to 0.008, 0.04, 0.2, 1, 5 mg/ml. Samples, positive control, sample + positive control and negative control were designed, tests according to manufacturer's instructions. As shown in supplementary material Table S1, the results showed that the endotoxin level was negative in PM_2.5_.

### 2.4 Hemodynamic measurement

Pulmonary valve and aortic valve peak blood flow were monitored continuously in anesthetized (isoflurane) animals using pulse wave (PW) Doppler ultrasound using the cardiovascular scan head MS-201 and Vevo 2100 Imaging System (FUJIFILM VisualSonics Inc., USA). A subsample (*n* = 3) of control group and the high dose group could be tested.

### 2.5 Histopathological analysis

Animals were anesthetized by 5% chloralhydrate (7 ml/kg bw) and perfused with saline *in situ* through the tip of the heart. The pulmonary tissues and thoracic aorta were fixed in 4% paraformaldehyde overnight, embedded in paraffin, sectioned at 5 μm thickness, stained with hematoxylin and eosin (H&E) or stained for fibrin in lungs with Martius Scarlet Blue (MSB) and examined with blind method by Pannoramic Digital Slide Scanner (3DHISTECH, Hungary) according to the standard techniques.

### 2.6 Immunohistochemistry

The expression of CD31, TM and TF were quantified after immunohistochemical staining of the thoracic aorta. Sections of thoracic aorta were incubated with CD31 (1: 500) (Novus Biologicals, USA), TF (1:1000) and TM (1:1000) antibodies (Abcam, UK) at 4 °C overnight, washed with PBS (pH 7.4) and then incubated with secondary antibody. Sections were treated with Goat Anti-Rabbit IgG H&L (HRP), were stopped with freshly configured DAB solution, and counterstained with hematoxylin. The areas of positive staining were recognized and quantified by blind method using Pannoramic Digital Slide Scanner (3DHISTECH, Hungary).

### 2.7 Inflammatory cytokines and adhesion molecules detection

The levels of inflammatory factors such as interleukin (IL)-6, IL-1β, monocyte chemotactic protein (MCP)-1 were assessed in serum (centrifuge, 3000r, 10 min) using a ‘Rat Inflammation’ kit (Luminex, eBioscience, USA). C-reactive protein (CRP) and adhesion molecules (VCAM-1, ICAM-1) were quantified by ELISA reagent kits (CUSABio, UK), following the manufacturer's instructions, and detecting absorbance value at 450 nm by microplate spectrophotometer (Synergy HTX Multi-Mode Reader, BioTek, USA).

### 2.8 Coagulation parameters analysis

Rats were anesthetized using 5% chloralhydrate and blood collecting from the abdominal aorta to measure complete platelet count and coagulation parameters (PT, APTT, TT and fibrinogen) by automated coagulation analyzer. ELISA reagent kits (CUSABio, UK), tests in accordance with manufacturer's instructions, analyzed the coagulation factors (TF, FXa and vWF), anticoagulant (TFPI, ATIII and TAT) and fibrinolysis factors (D-dimer and t-PA).

### 2.9 qRT-PCR analysis

TRIzol reagent (Invitrogen, Thermo Fisher Scientific, USA) was used to extract the total mRNA from the thoracic aorta, followed by purification with a RNeasy kit (Qiagen, Valencia, CA, USA) according to the manufacturer's manual. The cDNA was synthetized by M-MLV reverse transcription (Promega, USA). qPCR analysis and data collection were performed on an ABI 7900HT qPCR system. The relative expression of each gene of interest was normalized to the relative expression of GAPDH (B661204-0001, Shanghai Sangon Biotech, China). The reaction conditions were as follows: after 15 min at 95 °C, 40 cycles were performed at 95 °C for 10s with annealing temperatures of 60 °C and then 60 °C for 30s. The data were analyzed using the 2^−△△Ct^ method. Primer sequences are listed at Table 1.

**Table 1:** qRT-PCR primers.

Gene Name	Forward primer	Reverse primer
JAK1	GTCCCTGAAGCCTGAGAGTG	CGTCTTCCATGCAGATTCCT
JAK2	TTGGCAACAGACAAGTGGAG	TGGGAGCTGGTGCTTATCTT
STAT3	CAGCCAGCAAAGAGTCACAC	TTCGCAGGTTGTGCTGATAG
IL-6	CACAGAGGATACCACCCACA	CAGAATTGCCATTGCACAAC
IL-1β	TCTCACAGCAGCATCTCGAC	TCTCACAGCAGCATCTCGAC
TNF	TGCCTCAGCCTCTTCTCATT	ATGAGAGGGAGCCCATTTG
vWF	ACAATGGCAACAAGGGAGAC	GCTGTGTTGTCTGTGCAGGT
FX	CTGGGTAATGACGGCAAGTC	GGTCTTCAGGGTCAGGTTCA

### 2.10 Statistics analysis

All data are expressed as mean ± S.D. Statistical significance was evaluated using the unpaired Student's *t*-test or one-way analysis of variance (ANOVA) for sample comparison between experimental and control groups. Differences were considered statistically significant at *p* < 0.05.

## 3 Results

### 3.1 PM_2.5_ altered vascular hemodynamics

**Fig. 1:**
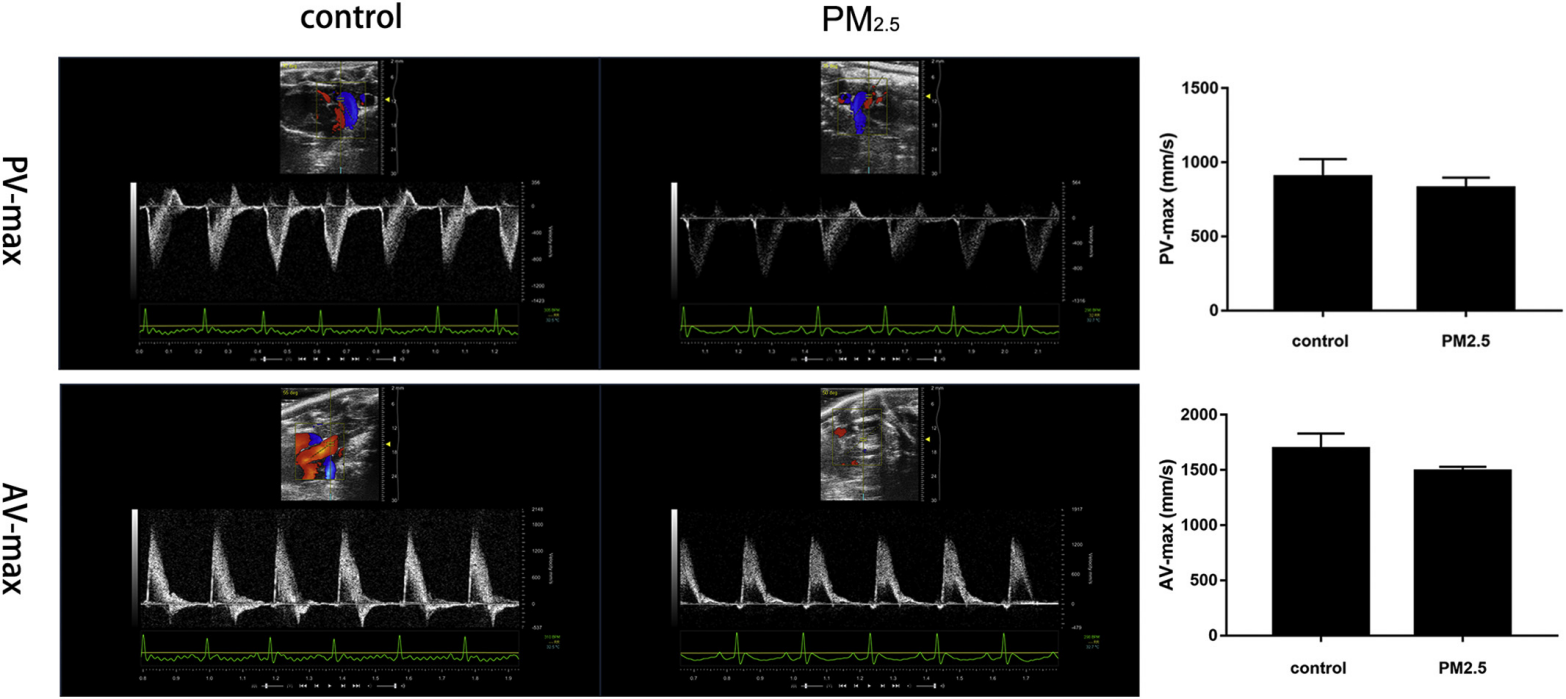
Alterations in the hemodynamic in rats treated with PM_2.5_. Both PV-max and AV-max were induced in PM_2.5_ group compared to control group (saline) but didn't achieve significance. Dates are expressed as means c ± S.D.

PW Doppler mode ultrasound was used to observe the effects of PM_2.5_ exposure on hemodynamic function in rats (Fig. 1). The pulmonary valve peak blood flow (PV-max) showed a decreasing trend in the high dose PM_2.5_ group compared with the control group (826 mm/s and 902 mm/s, respectively). In PM_2.5_-treated rats showed lower aortic valve peak blood flow (AV-max) than in that of control (1489 mm/s and 1695 mm/s, respectively). Our results indicated that exposure to PM_2.5_ might have the capacity to promote coagulation *in vivo*.

### 3.2 PM_2.5_ induced pathological changes in lung tissue and vessel

**Fig. 2:**
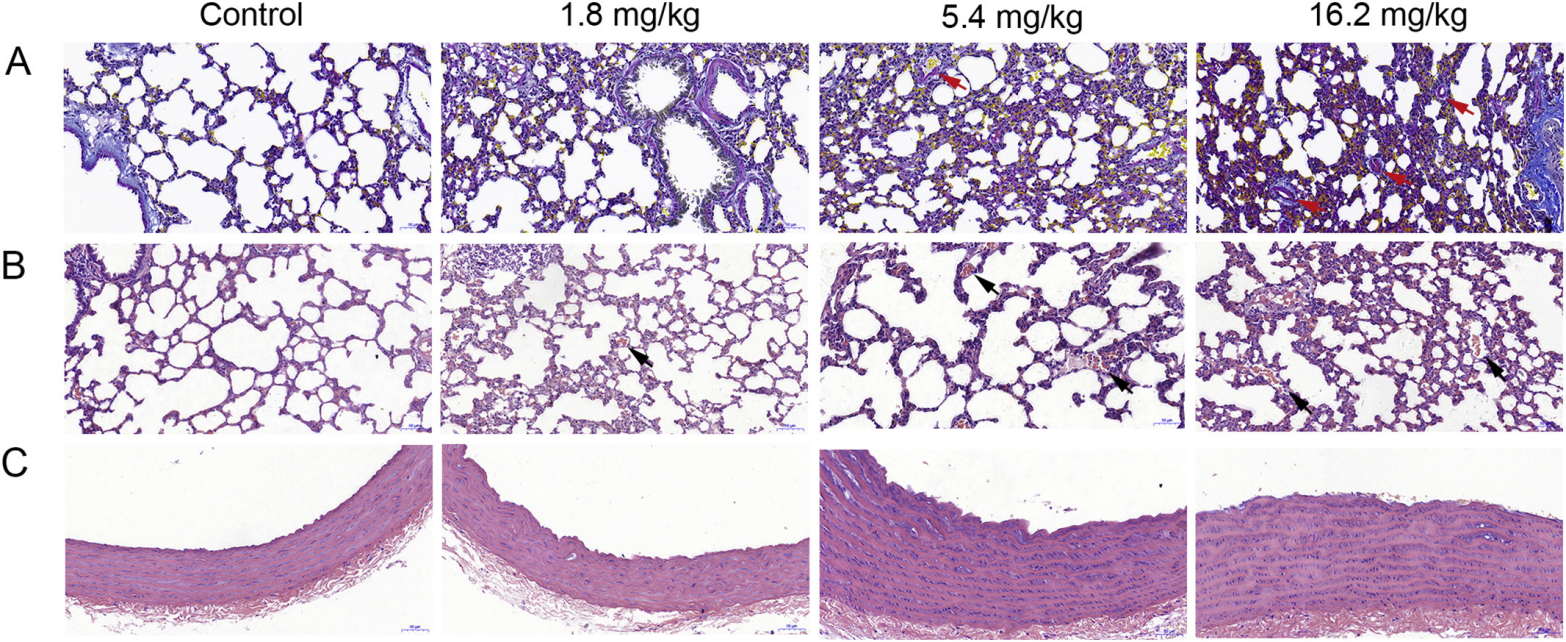
PM_2.5_ induced fibrin deposition in pulmonary arterioles (A), red arrows indicate positive staining of fibrin. Bleeding in the pulmonary alveoli of rats (B), black arrows indicate alveoli bleeding. And vascular endothelial injury (C). (For interpretation of the references to colour in this figure legend, the reader is referred to the web version of this article.)

Following PM_2.5_ exposure, the red homogeneity fibrin thrombi were observed in the pulmonary arterioles by the MSB staining (Fig. 2A). The alveolar wall thickening and erythrocytes exudation into the alveolar cavity in compared with the control (Fig. 2B). Relatively intact vascular endothelial layers were observed in control group and the low dose group of PM_2.5_, while it was difficult to observe in the middle and high dose groups (Fig. 2C). Thus, PM_2.5_ induced the phenomena of bleeding in lung tissue and vascular endothelial injury.

### 3.3 PM_2.5_ downregulated vascular CD31 and TM, and activated TF

**Fig. 3:**
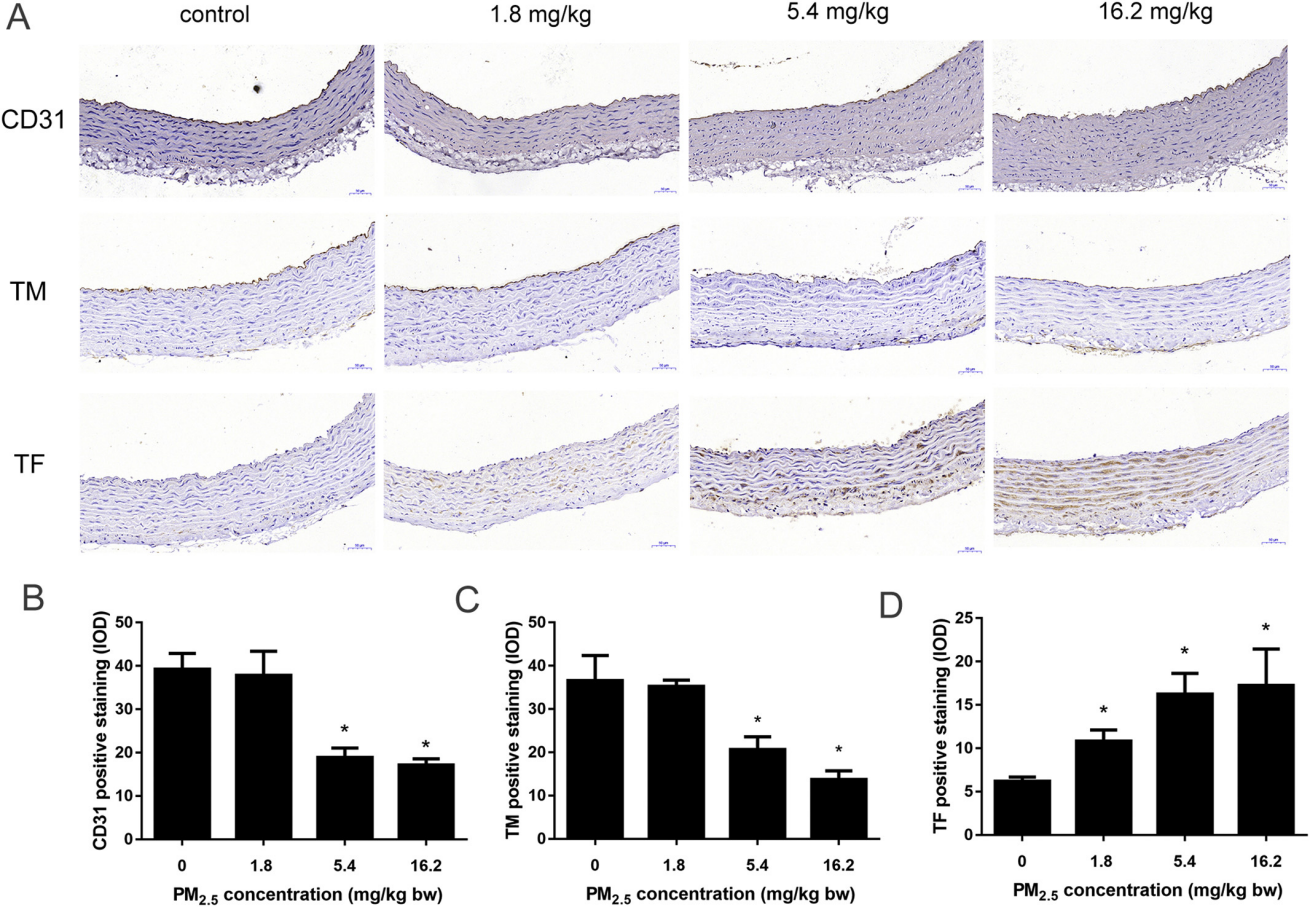
Immunohistochemical analyses of thoracic aorta after treated of rats with PM_2.5_ (A). (B, C) CD31 and Thrombomodulin (TM) were significantly lowered, (D) and tissue factor (TF) was markedly increased after exposure to PM_2.5_. Date are expressed as the means ± S.D., * *p* < 0.05.

CD31 and TM were mainly expressed in the vascular endothelial cells (Fig. 3). Levels were significantly downregulated in the PM_2.5_ groups compare to that of control group. In addition, there was significant activation of TF in PM_2.5_-treated groups, but not in the control group. These results support the findings that PM_2.5_ exposure led to vascular endothelial injury, in concert with alterations in coagulation factors.

### 3.4 PM_2.5_ increased proinflammatory cytokines and adhesion molecules in plasma

**Fig. 4:**
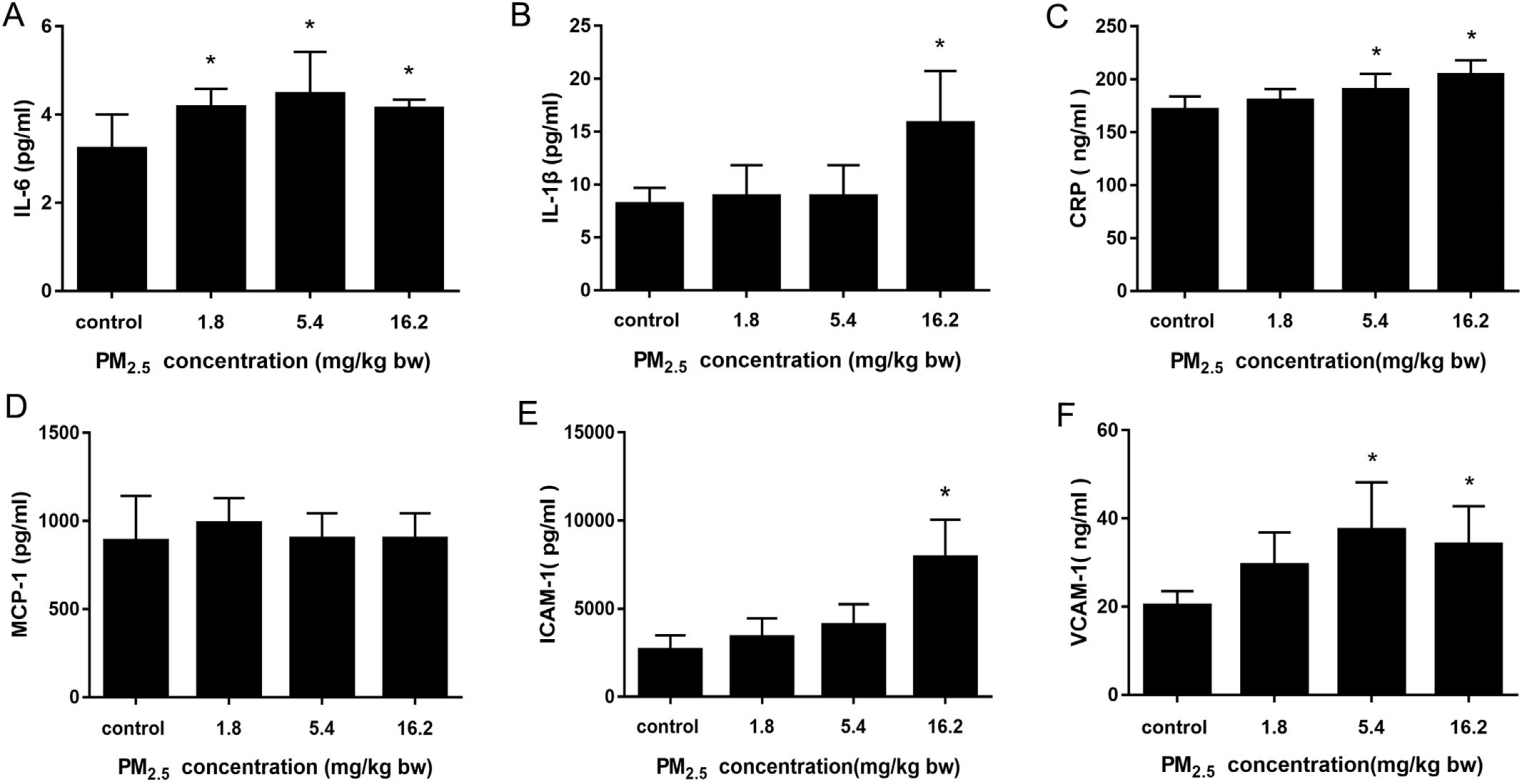
PM_2.5_ increased markers of inflammation in plasma. (A) IL-6; (B) IL-1β; (C) C-reactive protein (CRP); (D) Monocyte chemoattractant protein (MCP)-1 was non-significantly increased; (E) Intercellular adhesion molecule (ICAM)-1 and (F) vascular cell adhesion molecule (VCAM)-1 were markedly enhanced. Date are expressed as means ± S.D., * *p* < 0.05.

Expression of IL-6, IL-1β and CRP were increased in plasma in PM_2.5_-treated groups compared with saline-treated rats, whereas there was little effect on MCP-1(Fig. 4). Cellular adhesion molecules, VCAM-1 and ICAM-1, were markedly increased by 1.42- and 2.96-fold of the control, respectively, after exposure to the high-dose PM_2.5_. These results suggested that PM_2.5_ triggered a systemic inflammatory response.

### 3.5 PM_2.5_ activated the TF-dependent exogenous coagulation system

**Fig. 5:**
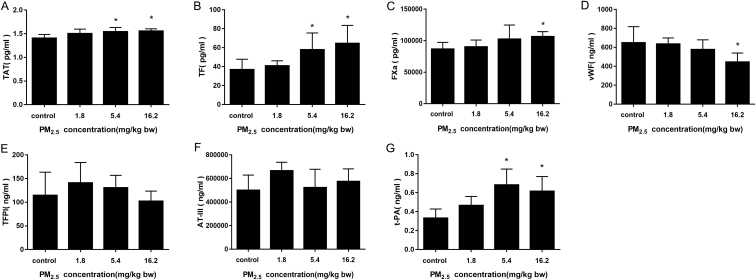
PM_2.5_ induced dysfunction of coagulation pathways in rats. (A) thrombin-antithrombin complex (TAT); (B) tissue factor (TF); (C) FXa; (D) vWF; (E) tissue factor pathway inhibitor (TFPI); (F) antithrombin III (AT-III); (G) tissue plasminogen activator (t-PA). Data are expressed as means ± S.D., * *p* < 0.05.

Serum levels of the thrombin-antithrombin complex (TAT) were upregulated markedly after PM_2.5_ exposure. TF increased significantly between the middle and high dosage groups of PM_2.5_, which were nearly 1.57 and 1.75-fold higher than the control group respectively. FXa in the high dose PM_2.5_ group was about 1.23 times higher than the control group, while levels of vWF were reduced. Our results (Fig. 5) demonstrated that PM_2.5_ induced hypercoagulability by activating the TF-dependent exogenous coagulation pathway *in vivo*.

### 3.6 PM_2.5_ activated the fibrinolytic system but did not absolutely activate the anticoagulant system

Under normal conditions, the coagulation system and fibrinolytic system are in dynamic equilibrium. We analyzed alterations in anticoagulant and fibrinolytic system after PM_2.5_ treatment (Fig. 5). The tissue factor pathway inhibitor (TFPI) and antithrombin III (AT-III) showed non-significantly change in PM_2.5_ treated rats compared with control group. In addition, level of tissue plasminogen activator (t-PA) was significantly increased. Together, our results suggested that PM_2.5_ treatment tends to partially promote the anticoagulant pathway, while activating the fibrinolytic system.

### 3.7 PM_2.5_ triggered the occurrence of DIC

**Fig. 6:**
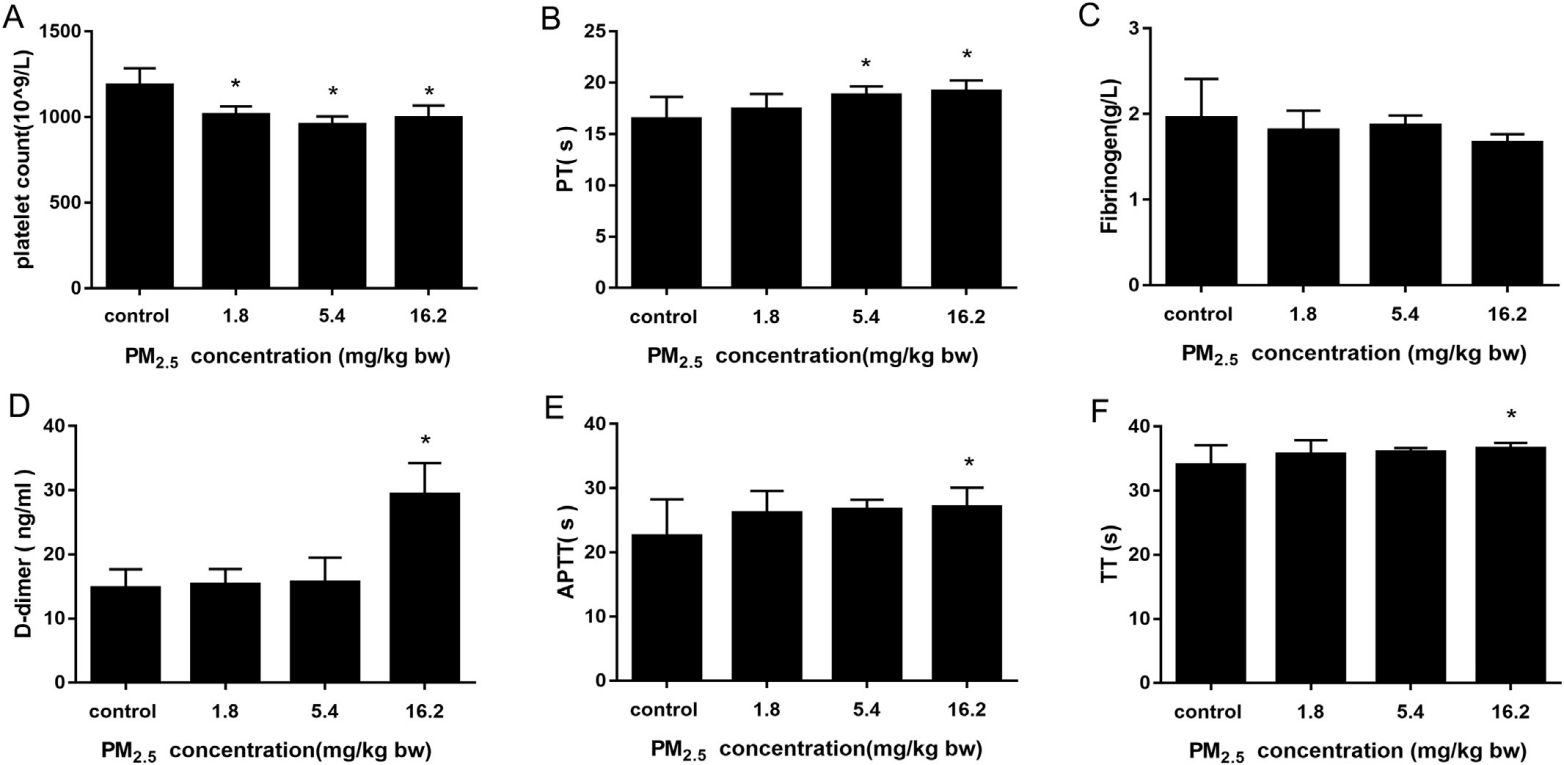
Induction of a DIC-like sate in rats treated with PM_2.5_, using the following indices: (A) the platelet count; (B) prothrombin time (PT); (C) fibrinogen; (D) D-dimer; (E) activated partial thromboplastin time (APTT); (F) thrombin time (TT). Date are expressed as the means ± S.D., * *p* < 0.05.

To clarify the relationship between PM_2.5_ and DIC, we examined the general diagnostic indices of DIC: platelet count, prothrombin time (PT), fibrinogen and D-dimer (Fig. 6). PM_2.5_ reduced platelet count and prolonged PT by approximately 2.7 s. Fibrinogen showed a non-significant decreasing trend. D-dimer was significantly increased nearly 2-fold in high PM_2.5_ treated rats compared with saline-treated rats. In addition, activated partial thromboplastin time (APTT) and thrombin time (TT) were prolonged significantly in highest dosage group of PM_2.5_. Overall, the pattern of response to PM_2.5_ on diagnostic induces suggests the occurrence of DIC in this model.

### 3.8 PM_2.5_ induced inflammation and hypercoagulability

**Fig. 7:**
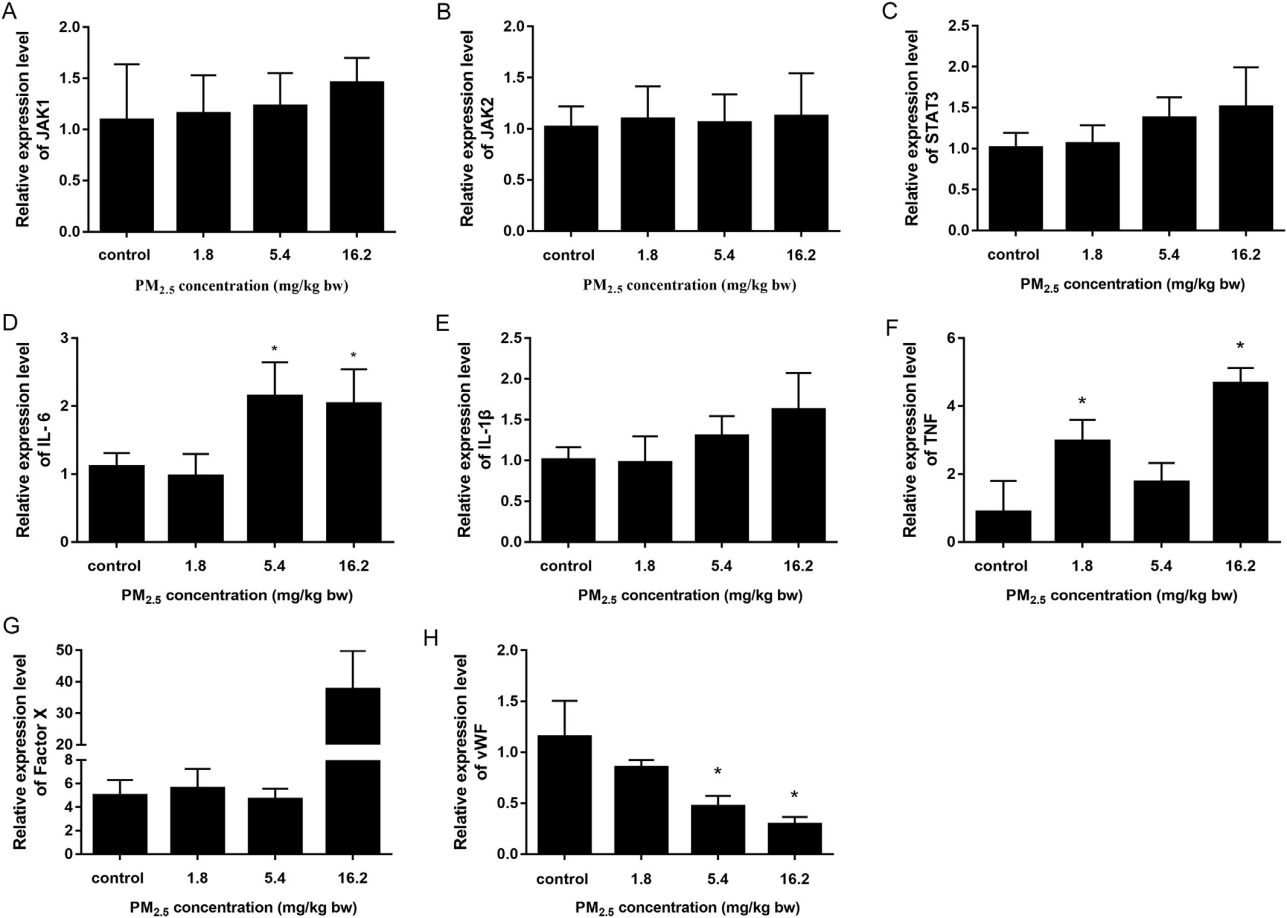
Aggravated vascular injury in rats following PM_2.5_ exposure. (A) mRNA expression for JAK1; (B) JAK2; (C) STAT3; (D) IL-6; (E) IL-1β; (F) TNF; (G) FX; (H) vWF. The data are presented as the means ± S.D., * *p* < 0.05.

qRT-PCR was used to gain a better understanding of the molecular mechanisms underlying the inflammatory and coagulatory effects of PM_2.5_ in blood vessels. The mRNA levels of JAK1 and STAT3 showed an upward trend and PM_2.5_ up-regulated the inflammation factors (IL-6, IL-1β and TNF), but had little effect on JAK2 (Fig. 7). The coagulation factor FX was also notably up-regulated. vWF was significantly downregulated in the PM_2.5_ treatment group compared with control group. Taken together, these data showed that PM_2.5_ triggered an inflammatory response and induced a state of hypercoagulability.

## 4 Discussion

Particulate matter in air pollution is a major cause of cardiovascular morbidity and mortality (Gold and Mittleman, 2013). Studies have found that long-term exposure to PM_2.5_ contribute to undesirable changes of coagulation and increase the incidence of deep venous thrombosis (Brook et al., 2010). DIC is an acquired syndrome characterized by widespread intravascular activation of coagulation. Animal studies showed that exposure to particulate matter induced inflammatory in lung, accelerated coagulation, disrupted fibrinolysis responses and accelerated arterial thrombus formation (Mutlu et al., 2007; Wu et al., 2012). However, it has not been reported that exposure to PM_2.5_ could trigger DIC. This is the first study to demonstrate that exposure to PM_2.5_ could trigger the DIC. The findings provide a new perspective by which inhaled PM_2.5_ could contribute to the cardiovascular mortality attributed to air pollution.

Disturbances of the microcirculation and abnormal haemodynamic properties are important factors that play an important role in DIC and result in organ dysfunction or failure (Niu et al., 2013). We did not note abnormities of hemodynamics in rats after exposure to PM_2.5_. Interestingly, we found fibrin positive staining and bleeding in pulmonary alveoli in rats following pulmonary exposure to urban PM_2.5_ from Beijing city center. DIC is a condition characterized by systemic activation of coagulation, causing intra- and extravascular fibrin deposition in kidneys, lungs, liver and brain potentially leading to thrombotic obstruction of micro vessels (Levi and Scully, 2018). Continuous over-activation of the coagulation system in pulmonary blood vessels could result in exhaustion of platelets and coagulation factors, leading to bleeding from these vessels (Levi, 2016). Moreover, activation of the fibrinolysis process leads to a high level of fibrin degradation products, which through the crosstalk between fibrin and platelets, can have an adverse effect on the hemostasis, potentially causing the bleeding (Levi and Scully, 2018). These evidences suggested that PM_2.5_ could contribute to the occurrence of DIC.

Inflammation remains a key pathway by which PM_2.5_ can induce endothelial cell dysfunction and vascular impairment (Pope et al., 2016). Studies have shown that a deficit in TM expression or function results in a greater propensity to develop inflammation and thrombosis (Ding et al., 2009). Early increases of TNF-α and IL-1β will down-regulate the level of TM in the endothelium in DIC patients after trauma (Gando and Otomo, 2015). Likewise, vascular endothelial injury contributes to the release of inflammatory factors and TF into the blood which activates both the endogenous and exogenous coagulation pathways (Foley and Conway, 2016). Our results concur with other studies showed that extensive crosstalk between inflammation and coagulation pathways leading to amplification of both pathways (Strukova, 2006). Overall, our results suggested that exposure to PM_2.5_ induces endothelial damage through inflammation, resulting in endothelial dysfunction *in vivo*.

TAT complexes were measured as a marker of thrombin generation marker, which could also mediate the PM_2.5_ induced hypercoagulability and increased the risk of thrombosis (Kalambokis et al., 2016). PM_2.5_ significantly activated TF, and other coagulation factors, leading to subsequent activation of the exogenous coagulation pathway. vWF is a co-mediator for during platelet aggregation at injured vessel walls. The down-regulation of vWF expression implied that vWF was consumed during the process of platelet aggregation (D. Zhang et al. 2017). Three other anticoagulant pathways (APC, TFPI and AT) also play important roles in regulating clot formation (Alhamdi and Toh, 2017), however, we did not observe that these anticoagulant systems were activated in PM_2.5_-treated rats in the present study. Continuous TF-dependent activation typically exhausts the availability of TFPI under disease conditions associated with DIC, leading to marked imbalance in coagulation and anticoagulant system (Osterud and Bjorklid, 2001). All physiological anticoagulant pathways are significantly compromised in DIC (Thachil, 2016). In addition, the upregulation of inflammatory factors and thrombin stimulate the synthesis and release of t-PA by vascular endothelial cells and which promotes secondary fibrinolysis (Fujie et al., 2017). Overall, exposure to PM_2.5_ contributed to the coagulation disorders.

The triggering of coagulation disorders could contribute to the development of many diseases. The most extreme form of systemic coagulation activation is seen as DIC (Levi, 2007). Epidemiological study showed that air pollution contributes to risk of deep vein thrombosis (Pope 3rd., 2009). After exposure to PM_2.5_, there was a prolongation of prothrombin time, platelet count decreased and fibrinogen consumed. Additionally, the fibrinolytic system was activated, increasing the level of D-dimer. Ongoing activation of platelets with consummation of coagulation factors consumption can contribute to the derangement of coagulation and fibrinolysis, leading to microvascular fibrin thrombi (Boral et al., 2016). Studies have also found that consumption of coagulation factors prolongs the PT and APTT in trauma patients with DIC (Gando and Otomo, 2015). These patterns of the molecular pathways in the present study suggest that PM_2.5_ can induce the derangement of coagulation and fibrinolysis causing DIC-like prothrombotic state.

The vascular endothelium plays an important role in regulating vascular tone and maintaining an anticoagulant and anti-inflammatory phenotype (Mudau et al., 2012). Inflammation was considered as an important exacerbating mechanism of coagulation disorders (Iba and Levy, 2018). Airborne-particulates can induce inflammatory response *via* activation of the TLR4/p38/NF-κB pathway (Li et al., 2017). Previous work of our group found that PM_2.5_ could induce the inflammatory response *via* activation of the JAK1/STAT3 signaling pathway *in vitro* (Hu et al., 2016). The present study doesn't show a mediating role for the JAK1/STAT3 pathways in promoting inflammation in the pro-coagulant effects of PM_2.5_ *in vivo*. The involvement of this pathway adds further support the close-interplay between inflammation and coagulation to provide the potential by which PM_2.5_ could induce DIC.

## 5 Conclusion

Using a rat model, we show that PM_2.5_ could induce inflammatory response, vascular endothelial injury leading to activate of TF-dependent coagulation, and prothrombotic state, eventually resulted in DIC. This study provides a new perspective on the means by which PM_2.5_ exposure induces the cardiovascular morbidity and mortality. The study adds further impetus for the urgent need to tackle the health effects of air pollution.

The following is the supplementary data related to this article.

Table S1 Endotoxin detection by TAL assay. Table S1
