# Supplementary material for: Repeat dose exposure of PM2.5 triggers the disseminated intravascular coagulation (DIC) in SD rats
Source: Sci Total Environ. 2019 May 1;663:245–53. doi: 10.1016/j.scitotenv.2019.01.346 (PMC6398278; doi:10.1016/j.scitotenv.2019.01.346)
Supplement: Table S1 — Endotoxin detection by TAL assay. [file mmc1.pdf]

Supplementary Information

Repeat dose exposure of $PM_{2.5}$ triggers the Disseminated Intravascular Coagulation (DIC) in SD Rats

Shuang Liang[1,2,#], Tong Zhao[1,2,#], Hejing Hu[1,2], Yanfeng Shi[1,2], Qing Xu[3], Mark R. Miller[4], Junchao Duan[1,2,] *, Zhiwei Sun[1,2,] *

[1] *Department of Toxicology and Sanitary Chemistry, School of Public Health, Capital Medical University, Beijing 100069, P.R. China*

[2] *Beijing Key Laboratory of Environmental Toxicology, Capital Medical University, Beijing 100069, P.R. China*

[3] *Core Facility Centre, Capital Medical University, Beijing 100069, P.R. China*

[4] *University/BHF Centre for Cardiovascular Science, Queens Medical Research Institute, The University of Edinburgh, Edinburgh, UK*

[#] *These authors contribute equally to this work.*

Table S1. Endotoxin detection by TAL assay

Dosage	Sample	Sample + Positive control	Positive control	Negative control
5 mg/ml	-	+	+	-
1 mg/ml	-	+	+	-
0.2 mg/ml	-	+	+	-
0.04 mg/ml	-	+	+	-
0.008 mg/ml	-	+	+	-
